# Measurement of Amiodarone Levels in the Breast Milk of a Japanese Woman With Peripartum Cardiomyopathy

**DOI:** 10.7759/cureus.89301

**Published:** 2025-08-03

**Authors:** Chiho Watanabe, Toru Wachi, Shoko Kamada, Susumu Kawano, Shin Taguchi

**Affiliations:** 1 Department of Clinical Pharmacy, Japanese Red Cross Society Akita Hospital, Akita, JPN; 2 Pharmacy, Pharma Mirai Co. Ltd. Co-creation Future Citizen Pharmacy, Niigata, JPN; 3 Department of Clinical Pharmacy, School of Pharmacy, Syujitsu University, Okayama, JPN

**Keywords:** amiodarone, japanese, mono-n-desethyl amiodarone, peripartum cardiomyopathy, transferable into milk

## Abstract

This study aimed to measure the concentrations of amiodarone (AM) and its active metabolite, mono-*N*-desethylamiodarone (DEA), in the breast milk of postpartum Japanese women treated with AM for ventricular tachycardia associated with peripartum cardiomyopathy and to conduct a follow-up study on the long-term growth and development of infants after resumption of breastfeeding. The patient was a 28-year-old Japanese woman with no underlying diseases who developed ventricular tachycardia and peripartum cardiomyopathy after giving birth. She was administered AM for three days via a combination of oral and intravenous administration. Breastfeeding began 35 days after the end of AM. Breast milk was collected 14 and 39 days after the end of AM, and its concentration in breast milk was measured using high-performance liquid chromatography. Furthermore, the growth and developmental test results of the twins were tracked for three years. Fourteen days after the end of AM administration, the AM concentration in breast milk was 111 ng/mL and the DEA concentration was 143 ng/mL. After 39 days, the AM concentration in breast milk was 11.8 ng/mL, and the DEA concentration in breast milk was 36.6 ng/mL. Studies have reported that pregnant women receiving AM are at risk of causing infant hypothyroidism and developmental delays due to the drug's high iodine content. Given the potential risks of invasive procedures and low milk intake, thyroid function testing was not conducted in the infant. Both twins were noted to be obese but had no clinical complications, and developmental evaluations showed no deficits. It was believed that resuming breastfeeding at that time was appropriate. Considering the risks associated with hypothyroidism, it is necessary to carefully determine the timing of breastfeeding initiation based on factors such as the mother's AM dosage, maternal blood concentration, and the infant's breast milk intake. However, it is considered acceptable for breastfeeding women who have received oral or intravenous AM for several days postpartum to breastfeed their infants.

## Introduction

Newly diagnosed cases of peripartum cardiomyopathy in Japan are estimated to be 50 per year, affecting about one in 15,000 births [1]. Countries such as the United States have a much higher incidence rate (1 in 1,000-3,000 births) than in Japan [1]. However, in recent years, the prevalence of pregnancy-related complications has increased owing to the advanced maternal age, an increase in multiple pregnancies due to the spread of regenerative medicine, and increased awareness of peripartum cardiomyopathy among medical professionals. Consequently, the number of patients with peripartum cardiomyopathy is increasing in developed countries [1]. An analysis of maternal mortality records by the Japan Society of Obstetrics and Gynecology reported that the cardiovascular disease-related deaths of three in 15 mothers between 2010 and 2012 were due to peripartum cardiomyopathy [2]. This suggests that this disease poses a threat to the lives of mothers in Japanese perinatal care.

Amiodarone (AM) is a Vaughan Williams class III antiarrhythmic drug that is widely used for ventricular arrhythmias because of its efficacy and low proarrhythmic effects [3]. In a chapter on arrhythmias during pregnancy, the Japanese Guidelines for Drug Therapy of Arrhythmias (2022 revised edition) stated that AM should be avoided during pregnancy because of its effects on the fetal thyroid gland. However, this restriction does not apply to patients with cardiac dysfunction or those at high risk [4]. Thus, AM is a drug that may be used during the perinatal period. While several studies highlight the risks of AM use during pregnancy, the reports on its long-term effects in children remain limited [5-11]. AM has a long half-life of approximately 55 days [12,13]. Considering that infancy is a critical period for thyroid function development [14,15], it is essential to evaluate the long-term effects of drug exposure during this stage. Therefore, this data is important for determining the timing of breastfeeding resumption in women who have been administered AM. Furthermore, there are no reports measuring breastmilk concentration of AM in patients with peripartum cardiomyopathy receiving AM intravenously and orally after childbirth, nor in Japanese women. Considering racial differences, lifestyle habits, and the increasing number of patients with peripartum cardiomyopathy, measurement data from Japanese women are useful.

In this study, we measured the concentration of AM in the milk of postpartum Japanese women who were administered AM intravenously and orally for three days to treat ventricular tachycardia associated with peripartum cardiomyopathy. In addition, we report the results of a three-year follow-up study on the growth of children.

## Case presentation

Ethical considerations

This research was conducted in accordance with the Ethical Guidelines for Medical and Health Research Involving Human Subjects and with the approval of the Akita Red Cross Hospital Ethics Review Committee (Approval No.: Akibyo Sogo No. 669, 768).

Breastfeeding mother

A 28-year-old Japanese woman, 159 cm tall, weighed 58.0 kg pre-pregnancy and 75.9 kg at admission. Her body mass index (BMI) was 22.9 kg/m² before pregnancy and 29.9 kg/m² at admission. She had a history of childhood asthma, but was not on any regular medications. Her obstetric history included one pregnancy and no prior births (G1P0).

**Figure 1:**
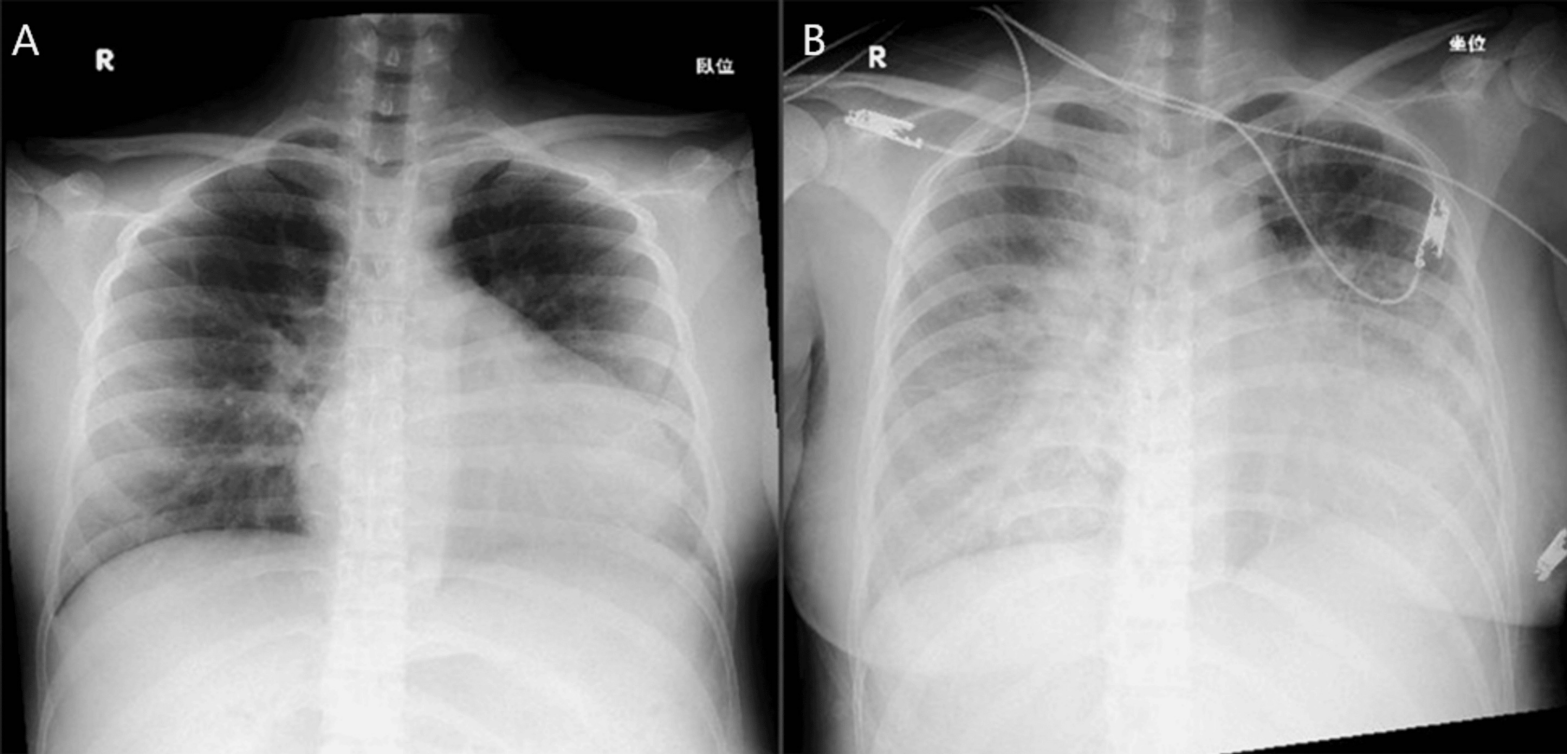
Chest X-ray of the mother A: At the time of admission; B: First day after giving birth

**Figure 2:**
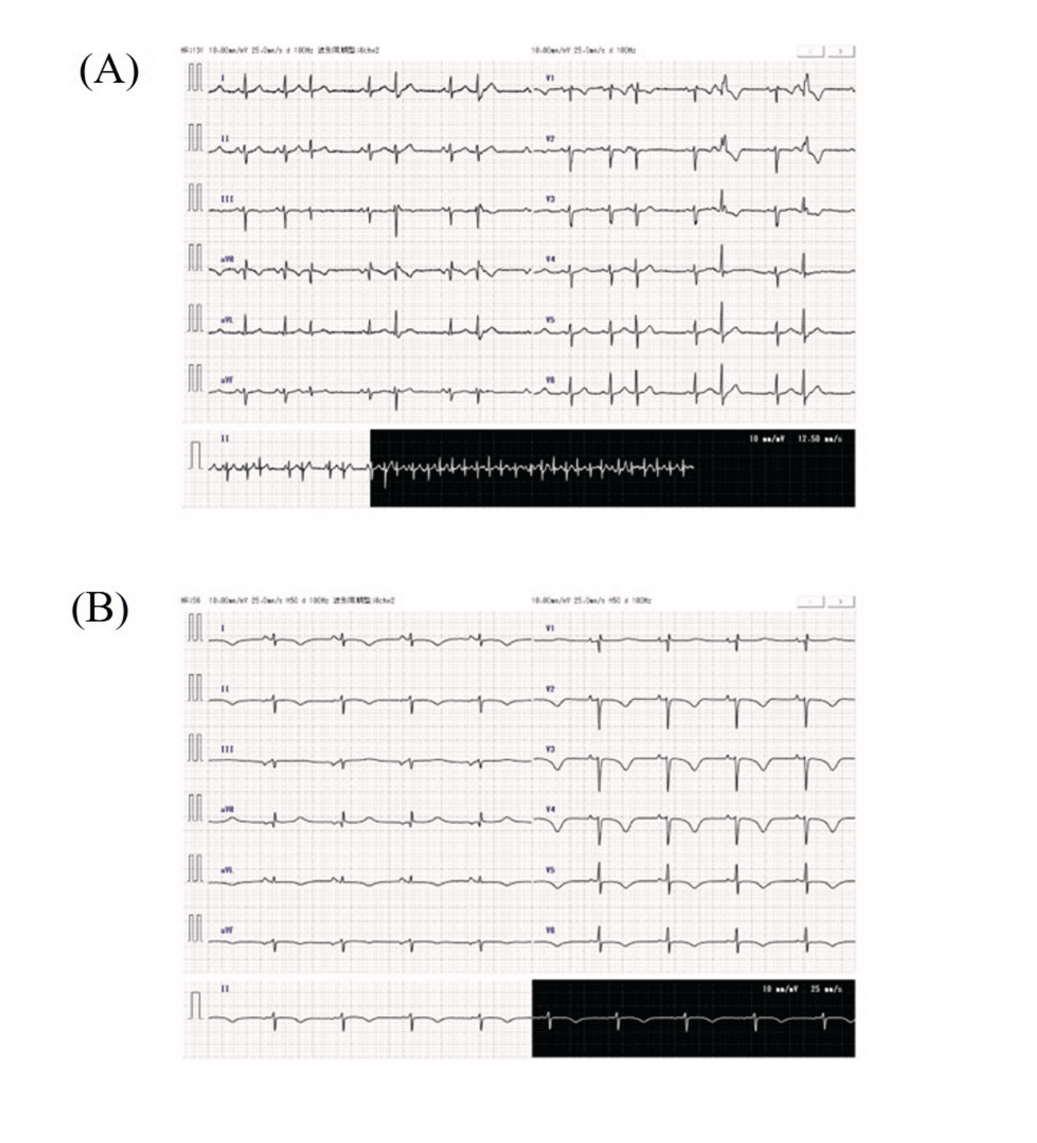
Twelve-lead electrocardiogram waveform of the mother A: At the time of admission; B: Four days after giving birth (when amiodarone treatment ended)

**Figure 3:**
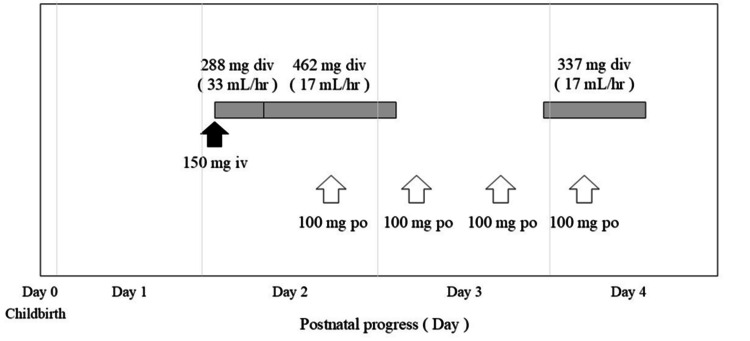
Dosage of amiodarone for the mother after childbirth The gray band at the top of the diagram indicates the intravenous infusion of amiodarone (AM). On the second day after birth, 150 mg AM was administered intravenously for 10 min (black arrow). Subsequently, 288 mg was administered at a rate of 33 mL/h as a loading dose, and the rate was reduced to a maintenance rate of 17 mL/h; 462 mg was administered for a total of 750 mg, at which point intravenous administration was completed. Arrhythmia recurrence was observed at the end of the third day after birth. AM administration was resumed at a rate of 17 mL/h, and intravenous administration was terminated after 337 mg was administered. The lower part of the figure shows oral administration of AM (white arrow). The mother started taking 100 mg orally once the second night after childbirth and completed four doses at 12-hour intervals.

**Figure 4:**
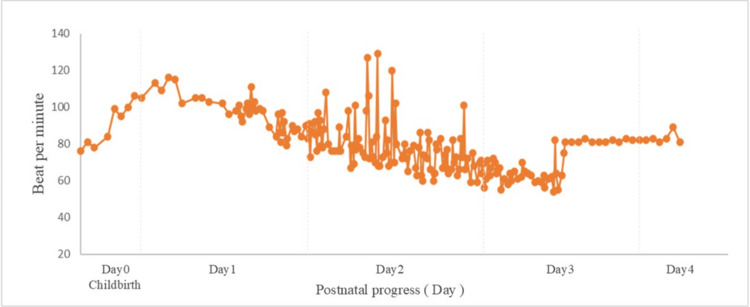
Pulse rate changes in the mother postpartum

**Figure 5:**
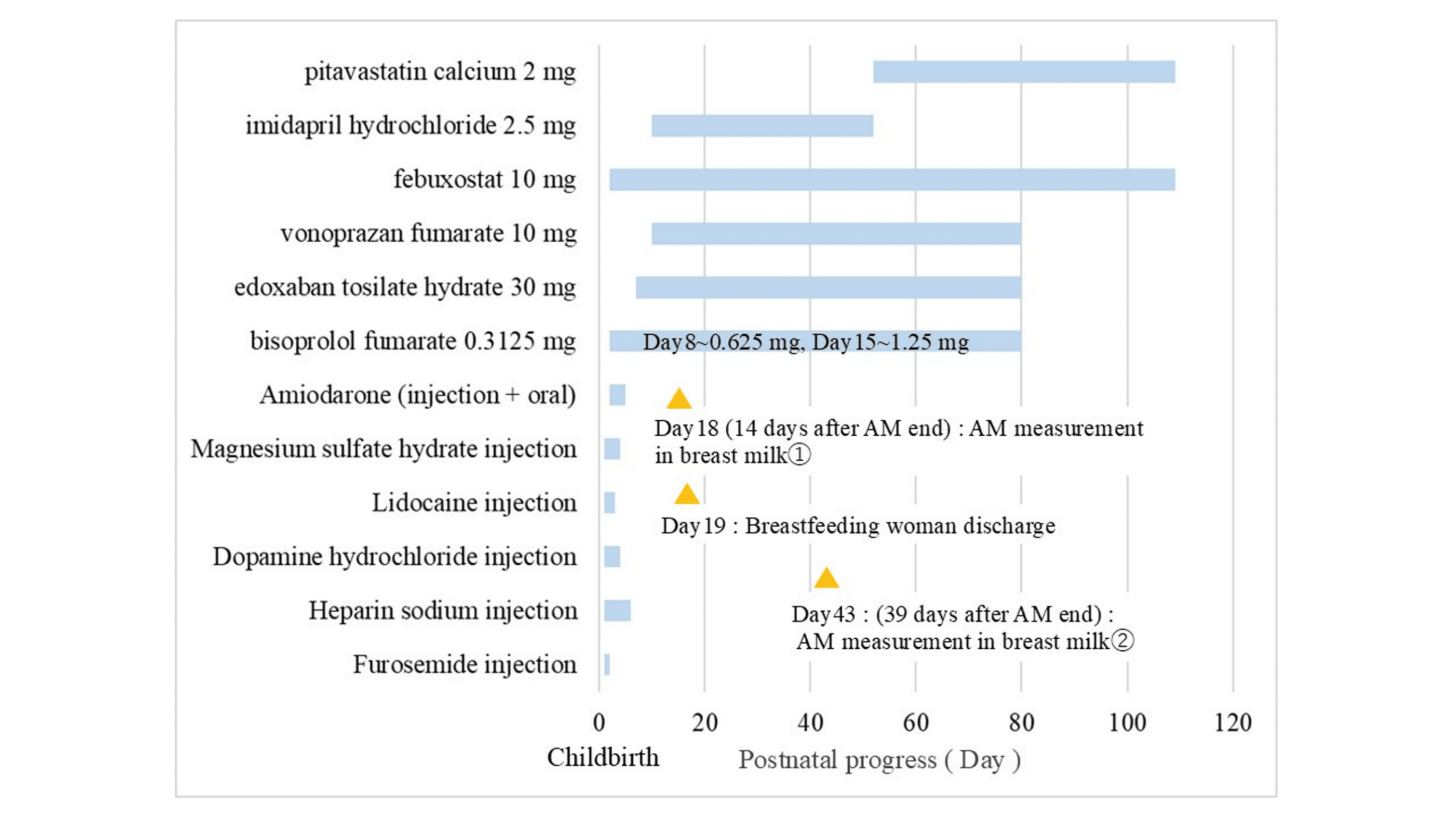
Timeline of medications administered to the mother during the perinatal period

Pregnancy was characterized by monochorionic diamniotic twin gestation. At 34 weeks and one day of pregnancy, the symptoms of cervical shortening worsened, and the patient was transferred to our hospital from the previous hospital. Upon admission, chest radiography revealed an enlarged heart (Figure 1A), and 12-lead electrocardiography revealed extrasystoles (Figure 2A). Therefore, the patient was referred to the cardiology department for evaluation of cardiac function. Echocardiography showed a left ventricular ejection fraction of 52.0%, which was at the lower limit of normal, but no other abnormalities were found; therefore, the patient was re-evaluated after childbirth. At 36 weeks and one day of pregnancy, occipito-pelvic disproportion and persistence of maternal arrhythmia were confirmed, and an emergency cesarean section was performed. On the first day after delivery, edema worsened, urine output decreased, orthopnea developed, and oxygen saturation decreased to 92%. The B-type natriuretic peptide (BNP) level increased to 990.9 pg/mL, and a simple chest radiograph showed bilateral pulmonary congestion and cardiac enlargement (Figure 1B); therefore, the patient was referred back to the cardiology department. Table 1 shows the results of the tests conducted on the mother at the time of admission and on the first day after delivery. The left ventricular ejection fraction was reduced to 12.0%. Peripartum cardiomyopathy and severe nonsustained ventricular tachycardia were diagnosed, and AM was initiated on the second day after birth. AM was administered from the second to the fourth day after childbirth. On the second day after birth, intravenous administration was initiated, and an external pacemaker was introduced in the evening of the same day; therefore, the administration method was switched to oral administration. However, no improvement in ventricular tachycardia was observed. Therefore, on the third day after birth, intravenous drip infusion and oral administration were combined. After taking the AM on the morning of the fourth day after birth, the patient showed improvement in ventricular tachycardia and prolongation of the QT interval on a 12-lead electrocardiogram (Figure 2B), and the continuous intravenous drip was terminated at noon. The AM dosage is shown in Figure 3, and the pulse rate changes in postpartum women are shown in Figure 4. In addition to AM, furosemide, heparin sodium, dopamine hydrochloride, lidocaine, and magnesium sulfate hydrate injections were administered. We started oral administration of bisoprolol fumarate 0.3125 mg, edoxaban tosilate hydrate 30 mg, vonoprazan fumarate 10 mg, febuxostat 10 mg, imidapril hydrochloride 2.5 mg, and pitavastatin calcium 2 mg. The bisoprolol fumarate dose was gradually increased to 1.25 mg. The patient continued to take oral medications after breastfeeding was initiated. Figure 5 shows the timeline of the medications used during the perinatal period. The woman remained hospitalized for 19 days after delivery (15 days after the end of AM administration), when her heart function stabilized.

**Table 1:** The mother's test results TP: total protein, Alb: Albumin, CRE: creatinine, eGFR: estimated glomerular filtration rate, UA: uric acid, LD: lactate dehydrogenase, AST: aspartate aminotransferase, ALT: alanine aminotransferase, CK: creatine kinase, CRP: c-reactive protein, HGB: hemoglobin, PLT: platelets

Inspection items	The time of admission	First day after birth
TP(d/dL)	5.8	4.8
Alb(g/dL)	3.1	2.3
CRE(mg/dL)	0.69	1.00
eGFR(mL/min/1.73m^2^)	84.5	56.3
UA(mg/dL)	8.6	11.3
Na(mmol/L)	140	137
Cl(mmol/L)	110	110
LD(U/L)	227	473
AST(U/L)	16	77
ALT(U/L)	11	41
CK(U/L)	70	1785
CRP(mg/dL)	0.38	8.01
WBC(10^2^/μL)	62	158
RBC(10^4^/μL)	343	389
HGB(g/dL)	9.6	11.5
PLT(10^4^/μL)	17.6	19.8
aortic diameter(cm)	2.8	2.8
left atrial diameter(cm)	3.2	4.1
left ventricular end-diastolic diameter(cm)	5.3	5.5
left ventricular end-systolic diameter(cm)	3.8	5.2
ejection fraction(%)	52.0	12.0

Newborn babies

The birth weight of the first child was 2,439 g, height 46 cm, and head circumference 34.5 cm. The second child was born weighing 1,503 g, with a height of 39.5 cm and a head circumference of 32 cm.

Sampling

On the 14th day after AM administration, we collected the mother’s blood and measured the concentrations of AM and mono-N-desethylamiodarone (DEA), an active AM metabolite.

Two samples (5 mL each) were collected 14 and 39 days after AM administration. As we did not have the facilities to measure the AM and DEA concentrations in the milk, we froze the samples at −20 °C and sent them to the Faculty of Pharmaceutical Sciences at Shujitsu University for analysis. A previous report showed that AM exhibits high solubility and stability under repeated freezing and thawing conditions [16].

Measurement of AM concentration in breast milk

To 500 μL of the sample, 25 μL of 10 μg/mL imipramine and 500 μL of 3% sulfosalicylic acid were added as internal standards, and the mixture was stirred to remove proteins. Next, 2 mL of diethyl ether was added, and the mixture was stirred and centrifuged. After centrifugation, 1.5 mL of the ether phase was accurately transferred to a separate tube and evaporated to dryness in a 50 °C block heater. To create a high-performance liquid chromatography (HPLC) sample, we added 100 μL of methanol to the residue. In HPLC, mobile phase A consisted of 100 mM CH3COONH4 buffer solution with a pH of 4.0, while mobile phase B was composed of a 1:1 mixture of acetonitrile and methanol. The mobile phases were mixed, consisting of 25% A and 75% B. The delivery system used was LC-10ADvp (Shimadzu, Kyoto, Japan), with a flow rate of 0.8 mL/min, and the detector was SPD-10 AV (Shimadzu), with detection at 254 nm. The column was an X Bridge C183 μm, φ4.6 mm × 150 mm (Waters, Milford, MA, USA).

The sensitivity of this HPLC system varied linearly from 2.5 to 0.039 µg/mL for AMD and DEA, with regression coefficients of R^2=0.9984 and R^2=9994, respectively.

The recoveries of these samples were greater than 99%, and peaks were identified using fraction chromatograms and retention times that excluded the AMD and DEA peaks.

Measurement results for AM and DEA concentrations in blood and milk

Fourteen days after AM administration, the AM and DEA concentrations in the mother’s blood were 96.9 ng/mL and 126 ng/mL, respectively, and that in breast milk were 111 ng/mL and 143 ng/mL, respectively. The AM concentration in breast milk 39 days after the end of AM administration was 11.8 ng/mL, and the DEA concentration in breast milk was 36.6 ng/mL. The half-lives of AM and DEA in breast milk are 7.8 and 12.6 days, respectively.

**Figure 6:**
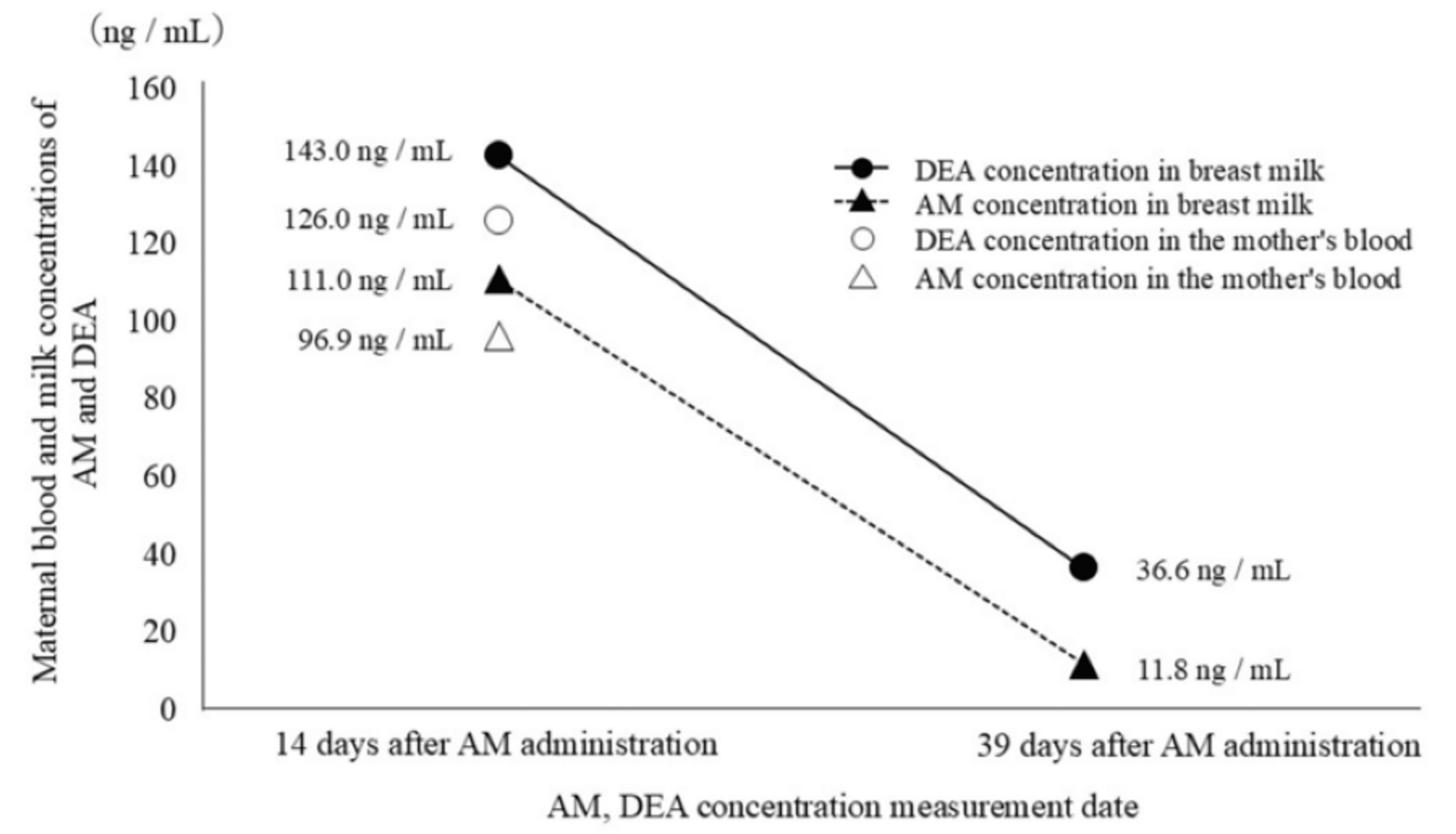
Results of measuring the maternal blood and milk concentrations of AM and DEA AM: amiodarone, DEA: mono-N-desethylamiodarone

The results of measuring the maternal blood and milk concentrations of AM and DEA are shown in Figure 6.

Progress of the newborns and results of physical measurements

Both twins were born with low birth weights and were therefore admitted to the neonatal intensive care unit. The first twin was hospitalized for 14 days, and the second twin for 32 days, until their weights had sufficiently increased. No other complications were observed. Fourteen days after the end of AM administration, the daily intake of infant formula was 450 mL for the first child and 350 mL for the second. Breastfeeding began 35 days after the end of AM administration. At that time, the daily intake of breast milk was 160 mL for the first child and 120 mL for the second. Breast milk was mixed with infant formula and accounted for approximately one-quarter of their total daily milk intake. At the start of breastfeeding, the first child weighed 3,360 g, and the second child weighed 2,174 g. The results of the twins' physical measurements from regular check-ups are shown in Table 2, and a summary of these measurements is presented in Table 3.

**Table 2:** Babies' physical measurements SD: Standard Deviation [17]

Lunar Age (month)	Weight (kg)		Height (cm)		Head circumference (cm)	
5	First child	8.2	First child	63.0	First child	43.8
	Second child	6.9	Second child	62.5	Second child	43.8
	±2SD range	5.45-8.71	±2SD range	60.0-68.0	±2SD range	38.8-43.8
11	First child	11.3	First child	74.1	First child	48.5
	Second child	10.0	Second child	72.0	Second child	48.5
	±2SD range	6.93-10.54	±2SD range	67.8-77.0	±2SD range	41.89-46.9
15	First child	12.1	First child	79.4	First child	49.7
	Second child	11.2	Second child	76.6	Second child	48.9
	±2SD range	7.62-11.44	±2SD range	71.9-81.4	±2SD range	42.89-48.14
26	First child	16.0	First child	88.7	First child	52.6
	Second child	14.0	Second child	86.5	Second child	51.3
	±2SD range	9.31-14.30	±2SD range	80.2-91.8	±2SD range	44.6-50.2

**Table 3:** Summary of babies' measurement data NICU: neonatal intensive care unit

Items	First child	Second child
Birth height	46cm	39.5cm
Birth weight	2439g	1503g
Birth diagnosis	Low birth weight infant	Low birth weight infant
NICU hospitalization period	14 days after birth	32 days after birth
Start of breastfeeding	39 days after birth	39 days after birth
Start breastfeeding weight	3360ｇ	2174g
Initial breast milk intake	160mL/day	120mL/day
Breastfeeding method	Mixed feeding	Mixed feeding
Three-year follow-up results （Health/Development）	Tendency toward obesity, no other complications/ no developmental delay	Tendency toward obesity, no other complications/ No developmental delay

## Discussion

Epidemiology of peripartum cardiomyopathy and the current situation in Japan

The incidence of peripartum cardiomyopathy varies greatly depending on the country, region, and ethnicity [1]. The incidence rate is the highest in Nigeria, with one case per 102 births, while in the United States, the incidence rate is one case per 1,000-3,000 births [1]. In contrast, Japan has the lowest incidence rate, with approximately one case per 15,000 births [1]. However, in recent years, the prevalence of pregnancy-related complications has increased owing to the advanced maternal age, an increase in multiple pregnancies due to the spread of regenerative medicine, and increased awareness of peripartum cardiomyopathy among medical professionals. Consequently, the number of patients with peripartum cardiomyopathy is increasing in developed countries [1]. According to a Japan Society of Obstetrics and Gynecology analysis of maternal mortality records (2010-2012), three of 15 cardiovascular disease-related deaths were due to peripartum cardiomyopathy [2], indicating that this disease poses a threat to the lives of mothers in Japanese perinatal care.

Maternal diagnosis

There are no internationally standardized diagnostic criteria for peripartum cardiomyopathy. However, the criteria proposed by Demakis et al. in 1971, with the addition of specific numerical values for left ventricular systolic dysfunction (left ventricular ejection fraction: LVEF ≤ 45%), are frequently used [18]. In Japan, clinical guidelines were published in 2019, stating that, women who develop new-onset cardiac dysfunction or heart failure (heart failure is not a mandatory criterion) between pregnancy and six months postpartum, with no other diseases that cause cardiac dysfunction or heart failure or history of heart disease prior to onset, had impaired left ventricular systolic function (LVEF ≤ 45%) [18]. This case presented with cardiac enlargement on a plain chest radiograph at 34 weeks of gestation (Figure 1A), premature contractions on a 12-lead electrocardiogram (Figure 2A), and on the first day after delivery, worsening edema, decreased urine output, orthopnea, a decrease in oxygen saturation to 92%, and an increase in BNP to 990.9 pg/mL. Chest radiography revealed bilateral pulmonary congestion and cardiac enlargement (Figure 1B). No other underlying diseases causing cardiac dysfunction or heart failure were identified, and there was no history of these conditions. On the first day after delivery, LVEF was found to have decreased to 12%, meeting the diagnostic criteria for peripartum cardiomyopathy, leading to the diagnosis.

Nonsustained ventricular tachycardia (NSVT) is generally defined as a series of three or more consecutive premature ventricular contractions with a cycle of 100 beats per minute or more [19]. Postpartum portable ECG data were not recorded in the electronic medical records and therefore cannot be shown as a graph; however, three or more premature ventricular contractions and a rapid heart rate of 100 beats per minute or more (Figure 4) were observed, leading to a diagnosis of NSVT.

Effects of therapeutic drugs on infants through breastfeeding

Treatment for peripartum cardiomyopathy is similar to that for general heart failure [20]. In addition, because this case involved ventricular tachycardia, treatment for arrhythmia was administered.

The effects of medications administered to postpartum women on their infants via breast milk, as well as the timing of breastfeeding initiation, were carefully considered. Furosemide, heparin sodium, dopamine hydrochloride, lidocaine, and magnesium sulfate hydrate were used only for a few days after childbirth and have relatively short half-lives. Since breastfeeding began 39 days after delivery, these medications were likely eliminated from the mother’s body by that time and were not expected to affect the infants. Therefore, it was determined that the oral medication in use could be continued during breastfeeding. This decision was based on reports indicating that bisoprolol fumarate transfers into breast milk only minimally to moderately and poses little risk to the infant [10]. Although there are no reports of febuxostat or pitavastatin calcium being detected in breast milk, both drugs exhibit plasma protein binding greater than 99%, which significantly limits their likelihood of being transferred into breast milk [21,22]. The plasma protein-binding rates of vonoprazan fumarate and imidapril hydrochloride are higher than 85% [23,24]; thus, the possibility of their transfer to breast milk is low. Imidapril hydrochloride has been reported to show low placental transfer and secretion into the breast milk in rats [25]. In contrast, no information is available on the use of edoxaban tosilate hydrate during breastfeeding in humans. The drug shows an in vitro plasma protein binding rate of 54.3-56.6% in humans, and studies in rats have demonstrated its transfer into breast milk [26]. Based on this, it was assumed that the patient was receiving a mixed-feed infant formula and that the transfer rate to the children was low; therefore, the treatment was initiated with the patient’s consent. While taking edoxaban tosilate hydrate, the mother breastfed her children for approximately two months after starting breastfeeding, and the children had no bleeding tendency.

The patient developed severe postpartum ventricular tachycardia, and treatment with dopamine hydrochloride, lidocaine hydrochloride, and magnesium sulfate injections did not improve her condition. Life-threatening arrhythmias, such as ventricular fibrillation and tachycardia, have been reported to increase the risk of sudden death [4,19,27]. Therefore, we prioritized maintaining the mother's life by using the AM until the external pacemaker was effective. Recommendation Table 67 of the Japanese Guidelines for the Management of Heart Failure recommends intravenous administration of nifecarant-AM to prevent the recurrence of ventricular tachycardia and fibrillation in patients with heart failure, with a recommendation class of IIa level of evidence (highly likely to be effective and useful based on evidence and opinion) [27]. On the contrary, administration of AM during pregnancy has been reported to increase the risk of hypothyroidism, goiter, and developmental delay in infants [7,9,28,29]. Since AM is fat-soluble, it transfers into breast milk, and takes time to be eliminated from the body [12]; therefore, the timing of breastfeeding resumption is a major concern. The mother in this case wished to breastfeed her baby; therefore, the AM concentration in her breast milk was measured to determine timing to initiate breastfeeding. Because our hospital initially lacked the equipment to measure AM concentrations in breast milk, we obtained consent to collect samples once the necessary equipment was available. The first sample was taken 14 days after AM administration ended. The second sample was collected 39 days after treatment completion, during the infant’s one-month checkup after the mother was discharged. This schedule was chosen to allow long-term evaluation, given AM’s half-life of approximately 55 days [12,13].

Although AM use during pregnancy has been reported in several studies [5-9], there are few reports on its use after childbirth [10,11]. When considering the timing of breastfeeding initiation, we referred to the studies by Khurana et al. [10] and Lucie et al. [11] on the use of AM administered intravenously after childbirth. A single AM dose of 150 mg [10], and 450 mg [11] administered intravenously to lactating women resulted in a low risk of infant exposure during lactation [10,11]. In Lucie et al.'s report, involving higher dose, the AM concentration in breast milk after 10 days was 132 µg/L. Although calculations based on pharmacokinetic considerations suggested that the risk to the infant through breastfeeding was low, AM was still detectable in breast milk after 10 days; therefore, as a precautionary measure to avoid exposure, breastfeeding was postponed for three weeks [11]. In this case, a single dose of AM did not improve the ventricular tachycardia; therefore, AM was administered for three days, including a single intravenous dose, followed by maintenance of intravenous and oral doses. The M/P ratio (drug concentration in breast milk/drug concentration in maternal plasma) was 1.15 ([AM concentration in breast milk 111 ng/mL] / [AM concentration in blood 96.9 ng/mL]) after 14 days, indicating that AM is concentrated in breast milk. Assuming that the first child ingested breast milk on the 14th day after birth, the daily drug dosage for the child would be approximately 0.05 mg/day, calculated from the breast milk AM concentration of 111 ng/mL × the child's daily milk intake of 450 mL/day. The Japanese guidelines for the diagnosis and treatment of pediatric arrhythmias recommend an initial dose of 10-20 mg/kg and a maintenance dose of 5-10 mg/kg for oral administration [30]. The estimated daily intake for children is approximately 0.05 mg/day, which is equivalent to 0.015 mg/kg for the firstborn child weighing 3.36 kg. Compared with the therapeutic dose for children, the intake is extremely low. However, because the child was an infant, there were concerns regarding thyroid dysfunction due to iodine accumulation during the developmental stage of the thyroid gland. In Lucie et al.'s report, the concentration in breast milk was 132 µg/L, which is equivalent to 132 ng/mL after 10 days. In our case, the AM concentration in breast milk 14 days after administration was detected at a concentration equivalent to that reported by Lucie et al. Based on existing reports, we administered AM intravenously and orally for three days, and considering the physical condition of the mother and child, we resumed breastfeeding 35 days after the end of AM administration. Breast milk was collected 39 days after the end of AM administration, and the AM concentration in breast milk was measured; however, the value was below the detection limit, suggesting that the infant's AM intake was negligible.

The mechanism of action of AM in hypothyroidism is that it contains high levels of iodine, which affects thyroid hormones during metabolism, and AM and DEA cause direct cell damage to thyroid cells [15,31]. In addition, thyroid hormones play a crucial role in the development of all organs, especially the brain [29]. Therefore, low thyroid hormone levels during the neonatal period can lead to delays in both mental and physical development [31]. Based on this, we decided that long-term observation of the child was necessary, and we tracked the results of regular checkups for three years after birth until the child was referred to a local doctor. Both twins were obese (Table 2), but had no complications and were considered to be in good health; therefore, no additional blood tests were performed. Because thyroid function tests were not conducted despite obesity, any causal relationship with AM is unclear; however, no thyroid enlargement was observed. Additionally, our hospital conducts an Enjoji-style Infant Analytical Developmental Test [32] during health checkups. In Japan, this test is commonly used as a simple tool to assess development across motor, social, and language skills [32]. The developmental test results revealed no abnormalities. The thyroid gland develops throughout the period from the fetal stage to infancy [14,15]. Reports of developmental delays caused by AM indicate that exposure occurred during pregnancy [6,7,28,29]. In this case, exposure to AM through breastfeeding began 39 days after delivery, and since there was no exposure during the critical period of fetal thyroid function development, it is likely that there were no problems with developmental testing. These findings imply that the resumption of breastfeeding was not too early in this case. Since the amount of AM exposure through breast milk was considered low, thyroid function tests were not performed. However, in cases where AM is administered during pregnancy or where the amount of AM exposure to the infant is expected to be high, thyroid function tests are necessary due to reports of hypothyroidism and goiter [6,7,28,29].

Half-life of AM in breast milk

AM has a long half-life, approximately 55 days [12,13]; however, in this case, the half-life of AM in breast milk was shorter than its serum half-life. We believe that the drug characteristics of AM were a factor in this. AM is highly lipophilic and has a high affinity for tissues; because it has a large distribution volume, it accumulates in fat tissue, the liver, and the lungs at relatively high concentrations [13]. Drugs with large distribution volumes tend to have lower blood concentrations [33]. There are also reports that when a woman becomes pregnant, body fat increases and is distributed across different parts of the body; overall, the amount of fat in the mother’s body increases [34], and women who are thin accumulate more fat during pregnancy than those with obesity [35]. Furthermore, a study that analyzed a Japanese population that had been taking AM orally for a long period reported that AM clearance was significantly higher in women than in men [36]. The target population for this analysis was older adults, with the average age of women being 72 and that of men being 75. At this age, women have significantly higher body fat than men, which may explain why clearance increases owing to the distribution of AM in adipose tissue [36]. In this case, the BMI before pregnancy was 22.9, which was within the normal weight range; however, the weight increased by 17.9 kg during pregnancy. Therefore, the amount of body fat might have increased owing to pregnancy. Since the administration of AM was short-term, lasting only three days, we believe that the distribution of AM in the adipose tissue increased, resulting in a lower blood concentration and lower transfer to breast milk.

Limitations

There are five limitations to this study. First, this was a single-case study, and data accumulation from similar cases is desirable. Second, thyroid function tests were not performed on the children. Although we suggested that the doctor perform the tests, they were not carried out because of the risk of invasion to the child and low breast milk intake. If a high AM intake is expected, thyroid function tests should be performed to assess the risk of hypothyroidism. Third, children’s developmental function testing was limited to indirect evaluations using the Enjoji Developmental Test. More detailed tests should be conducted in the future. Fourth, blood AM concentrations were measured 14 days after AM administration. Given that AM exhibits a three-compartment elimination profile [12] and has a long half-life of 55 days [12], blood concentrations should have been measured before and 14 days after AM administration. Fifth, the AM intake via breast milk in infants remains unknown. Breast milk intake after discharge was based on reports from mothers, and it was not possible to calculate the detailed AM intake of infants.

## Conclusions

This study measured the concentrations of AM and its active metabolite, DEA, in the breast milk of postpartum Japanese women who received a combination of intravenous and oral AM for three days after giving birth for ventricular tachycardia associated with peripartum cardiomyopathy and investigated the growth and development of infants after resuming breastfeeding for three years. Fourteen days after the end of AM administration, its concentration in breast milk was 111 ng/mL and the DEA concentration was 143 ng/mL. After 39 days, the AM and DEA concentration in breast milk was 11.8 ng/mL and 36.6 ng/mL, respectively. Because of the low intake of breast milk due to mixed feeding of breast milk and infant formula, breastfeeding of the twins resumed 35 days after the end of AM administration (39 days after delivery). Three-year follow-up study on the growth of newborns revealed obesity, but no health or developmental problems were observed. Considering the risk of hypothyroidism caused by AM, it is necessary to carefully determine the timing of breastfeeding based on factors such as the mother's AM dosage, blood concentration, period of exposure to AM, and amount of breast milk intake. Based on these data, it is considered possible for breastfeeding mothers who used AM for several days after giving birth to breastfeed their infants. In addition, when administering AM to pregnant or breastfeeding women, it is important to provide appropriate explanations regarding the necessity of administration and its effects on the child and to perform thyroid function tests on the child if exposure to AM is expected to be high.

## References

[1] Isogai T, Kamiya CA (2019). Worldwide incidence of peripartum cardiomyopathy and overall maternal mortality. Int Heart J.

[2] Tanaka H, Katsuragi S, Osato K (2017). The increase in the rate of maternal deaths related to cardiovascular disease in Japan from 1991-1992 to 2010-2012. J Cardiol.

[3] Pannone L, D'Angelo G, Gulletta S (2021). Amiodarone in ventricular arrhythmias: still a valuable resource?. Rev Cardiovasc Med.

[4] Ono K, Iwasaki YK, Akao M (2022). JCS/JHRS 2020 guideline on pharmacotherapy of cardiac arrhythmias. Circ J.

[5] Plomp TA, Vulsma T, Vijlder JJ (1992). Use of amiodarone during pregnancy. Eur J Obstet Gynecol Reprod Biol.

[6] Strunge P, Frandsen J, Andreasen F (1988). Amiodarone during pregnancy. Eur Heart J.

[7] Bartalena L, Bogazzi F, Braverman LE, Martino E (2001). Effects of amiodarone administration during pregnancy on neonatal thyroid function and subsequent neurodevelopment. J Endocrinol Invest.

[8] Hall CM, McCormick KP (2003). Amiodarone and breast feeding. Arch Dis Child Fetal Neonatal Ed.

[9] Basaria S, Cooper DS (2005). Amiodarone and the thyroid. Am J Med.

[10] Khurana R, Bin Jardan YA, Wilkie J, Brocks DR (2014). Breast milk concentrations of amiodarone, desethylamiodarone, and bisoprolol following short-term drug exposure: two case reports. J Clin Pharmacol.

[11] Javot L, Pape E, Yéléhé-Okouma M (2019). Intravenous single administration of amiodarone and breastfeeding. Fundam Clin Pharmacol.

[12] (2025). Pharmaceutical interview form, amiodarone (2024) [In Japanese]. https://med.toaeiyo.co.jp/products/amiodaroneinj/pdf/if-amzi.pdf.

[13] van Erven L, Schalij MJ (2010). Amiodarone: an effective antiarrhythmic drug with unusual side effects. Heart.

[14] Obregon MJ, Calvo RM, Escobar Del Rey F, Morreale de Escobar G (2007). Ontogenesis of thyroid function and interactions with maternal function. Endocr Dev.

[15] Eng L, Lam L (2020). Thyroid function during the fetal and neonatal periods. Neoreviews.

[16] Yamada Y, Mori K, Yamaguti K (2018). Development of the generic amiodarone injection which can be stored at room temperature for three years. https://www.shinryo-to-shinyaku.com/db/pdf/sin_0055_05_0361.pdf.

[17] (2025). Overview of the 2010 Infant and Toddler Physical Development Survey [In Japanese]. https://www.mhlw.go.jp/stf/houdou/0000042861.html.

[18] Kamiya C (2017). Diagnosis and treatment of peripartum. Circ J.

[19] Takase B, Ikeda T, Shimizu W (2024). JCS/JHRS 2022 Guideline on diagnosis and risk assessment of arrhythmia. Circ J.

[20] (2025). JCS 2018 Guideline on indication and management of pregnancy and delivery in women with heart disease [In Japanese]. https://www.j-circ.or.jp/cms/wp-content/uploads/2018/06/JCS2018_akagi_ikeda.pdf.

[21] (2025). Febuxostat. Drugs and Lactation Database (LactMed®) [Internet].

[22] (2025). Pitavastatin. Drugs and Lactation Database (LactMed®) [Internet].

[23] (2025). Pharmaceutical Interview Form, Takecab (2024) [In Japanese]. https://pins.japic.or.jp/pdf/medical_interview/IF00006509.pdf.

[24] (2025). Pharmaceutical Interview Form, Tanatril. (2025). [In Japanese]. https://pins.japic.or.jp/pdf/medical_interview/IF00000561.pdf.

[25] Endo M, Yamada Y, Kohno M, Suzuki T, Otsuka M, Takaiti O (1992). Metabolic fate of the new angiotensin-converting enzyme inhibitor imidapril in animals. 4th communication: placental transfer and secretion into milk in rats. Arzneimittelforschung.

[26] (2025). Pharmaceutical Interview Form, Lixiana. (2024). [In Japanese]. https://pins.japic.or.jp/pdf/medical_interview/IF00009303.pdf.

[27] Kitai T, Kohsaka S, Kato T (2025). JCS/JHFS 2025 Guideline on diagnosis and treatment of heart failure. Circ J.

[28] Ovadia M, Brito M, Hoyer GL, Marcus FI (1994). Human experience with amiodarone in the embryonic period. Am J Cardiol.

[29] Magee La, Downar E, Sermer M, Boulton BC, Allen LC, Koren G (1995). Pregnancy outcome after gestational exposure to amiodarone in Canada. Am J Obstet Gynecol.

[30] Naokata S, Mari I, Daichi U (2025). Guidelines for the Diagnosis and Treatment of Pediatric Arrhythmias [In Japanese]. https://jspccs.jp/wp-content/uploads/guideline_cure.pdf.

[31] Ylli D, Wartofsky L, Burman KD (2021). Evaluation and treatment of amiodarone-induced thyroid disorders. J Clin Endocrinol Metab.

[32] Kurokawa T (2021). About the Enjoji method of infant development analysis. Hist Pediatr Neurol.

[33] Smith DA, Beaumont K, Maurer TS, Di L (2015). Volume of distribution in drug design. J Med Chem.

[34] Pipe NG, Smith T, Halliday D, Edmonds CJ, Williams C, Coltart TM (1979). Changes in fat, fat-free mass and body water in human normal pregnancy. Br J Obstet Gynaecol.

[35] Maple-Brown LJ, Roman NM, Thomas A, Presley LH, Catalano PM (2013). Perinatal factors relating to changes in maternal body fat in late gestation. J Perinatol.

[36] Kashima A, Funahashi M, Fukumoto K, Komamura K, Kamakura S, Kitakaze M, Ueno K (2005). Pharmacokinetic characteristics of amiodarone in long-term oral therapy in Japanese population. Biol Pharm Bull.

